# CNN6mA: Interpretable neural network model based on position-specific CNN and cross-interactive network for 6mA site prediction

**DOI:** 10.1016/j.csbj.2022.12.043

**Published:** 2022-12-28

**Authors:** Sho Tsukiyama, Md Mehedi Hasan, Hiroyuki Kurata

**Affiliations:** a Department of Bioscience and Bioinformatics, Kyushu Institute of Technology, 680–4 Kawazu, Iizuka, Fukuoka 820-8502, Japan; b Tulane Center for Aging and Department of Medicine, Tulane University Health Sciences Center, New Orleans, LA 70112, USA

**Keywords:** N6-methyladenine, DNA modification, Deep learning, Machine learning, CNN, Interpretable prediction, 6mA, N6-methyladenine, SMRT, Single-molecule real-time, RF, Random forest, CNN, Convolutional neural network, LSTM, Long short-term memory, BERT, Bidirectional Encoder Representations from Transformers, AUCs, Area under the curves, SN, Sensitivity, SP, Specificity, MCC, Matthews correlation coefficient, t-SNE, t-distributed stochastic neighbor embedding, UMAP, Uniform manifold approximation and projection

## Abstract

N6-methyladenine (6mA) plays a critical role in various epigenetic processing including DNA replication, DNA repair, silencing, transcription, and diseases such as cancer. To understand such epigenetic mechanisms, 6 mA has been detected by high-throughput technologies on a genome-wide scale at single-base resolution, together with conventional methods such as immunoprecipitation, mass spectrometry and capillary electrophoresis, but these experimental approaches are time-consuming and laborious. To complement these problems, we have developed a CNN-based 6 mA site predictor, named CNN6mA, which proposed two new architectures: a position-specific 1-D convolutional layer and a cross-interactive network. In the position-specific 1-D convolutional layer, position-specific filters with different window sizes were applied to an inquiry sequence instead of sharing the same filters over all positions in order to extract the position-specific features at different levels. The cross-interactive network explored the relationships between all the nucleotide patterns within the inquiry sequence. Consequently, CNN6mA outperformed the existing state-of-the-art models in many species and created the contribution score vector that intelligibly interpret the prediction mechanism. The source codes and web application in CNN6mA are freely accessible at https://github.com/kuratahiroyuki/CNN6mA.git and http://kurata35.bio.kyutech.ac.jp/CNN6mA/, respectively.

## Graphical Abstract

**Figure:**
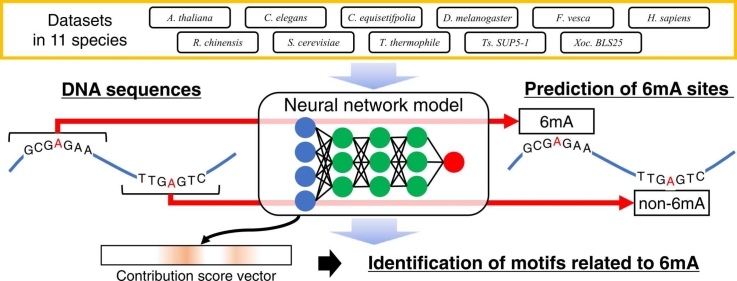

## 1 Introduction

N6-methyladenine (6 mA) is one of the epigenetic modifications which regulate genetic activities without any change of DNA sequences [1]. Recent studies present that N6-methyladenine (6 mA) plays vital roles in a variety of epigenetic processing including DNA replication [2], DNA repair [3], [4], silencing [5], transcription [6], and gene expression [7], [8], and enhances its significance in biological functions and diseases such as cancer [9]. To understand such epigenetic mechanisms, 6 mA has been detected by several experimental methods. Especially, high-throughput technologies including single-molecule real-time (SMRT) sequencing have been used for identification of 6 mA sites on a genome-wide scale at single-base resolution [10], [11], [12], together with conventional methods such as liquid chromatography-tandem mass spectrometry [13] and capillary electrophoresis with laser-induced fluorescence [14]. The validation with different experimental methods enables the identification of DNA modifications with high sensitivity and specificity, while these experimental approaches are time-consuming and laborious. Furthermore, some approaches are poor in the quality of sequencing and signal-to-noise ratios [15]. To complement the experimental methods and accelerate an understanding of 6 mA regulation mechanisms, *in silico* approaches of machine learning and deep learning have been developed.

Lv et al. proposed a random forest (RF) model called iDNA-MS [16] to predict 6 mA sites with the benchmark datasets of 11 species. This method showed high performances with learning small datasets, while there is room for improvement in the learning abilities with large datasets. To overcome this issue, convolutional neural network (CNN) and recurrent neural network including SICD6mA [17] and SNNRice6mA [18] have been developed, which predicted 6 mA sites with an accuracy (ACC) of more than 0.9 by capturing context information. Li et al. developed the Deep6mA [19] that combines CNN with Long Short-Term Memory (LSTM), which presented higher performances than SNNRice6mA [18] and MM-6mAPred [20]. The Deep6mA used CNN and LSTM to extract nucleotide patterns and to capture their context relationships, respectively, outperforming the CNN stand-alone model.

In addition to CNN and LSTM, an attention mechanism, which is incorporated in recent powerful neural network models such as Transformer [21] and Bidirectional Encoder Representations from Transformers (BERT) [22], has achieved remarkable improvements in the development of neural networks. Huang et al. combined LSTM with attention mechanisms and presented comparable performances to SICD6mA in 6 mA site prediction [23]. Yu et al. constructed the BERT-based neural network model, named iDNA-ABT [24], and compared it with the previous models including iDNA-MS and SNNRice6mA on the benchmark datasets constructed by Lv et al. (iDNA-MS). The BERT-based neural network generated the feature vectors, depending on the context information to extract different information exhaustively. iDNA-ABT presented higher ACCs and area under the curves (AUCs) in some species than the previous modification sites prediction models including iDNA-MS, DeepTorrent [25], and SNNRice6mA.

The aforementioned approaches accurately predicted 6 mA sites by using machine learning and deep learning, while they did not intensively address their prediction mechanism. In 2022, we have proposed BERT6mA [26] which combined BERT with word2vec-based encoding methods. BERT6mA presented the state-of-the-art performance by capturing the relationship between nucleotides at different positions and generated the attention weights to extract the features responsible for identifying 6 mA-associated nucleotide distributions. On the other hand, BERT6mA ignored the positional information of each nucleotide, while some studies regarding DNA and RNA modification prediction suggested the importance of position-specific features [27], [28].

**Fig. 1:**
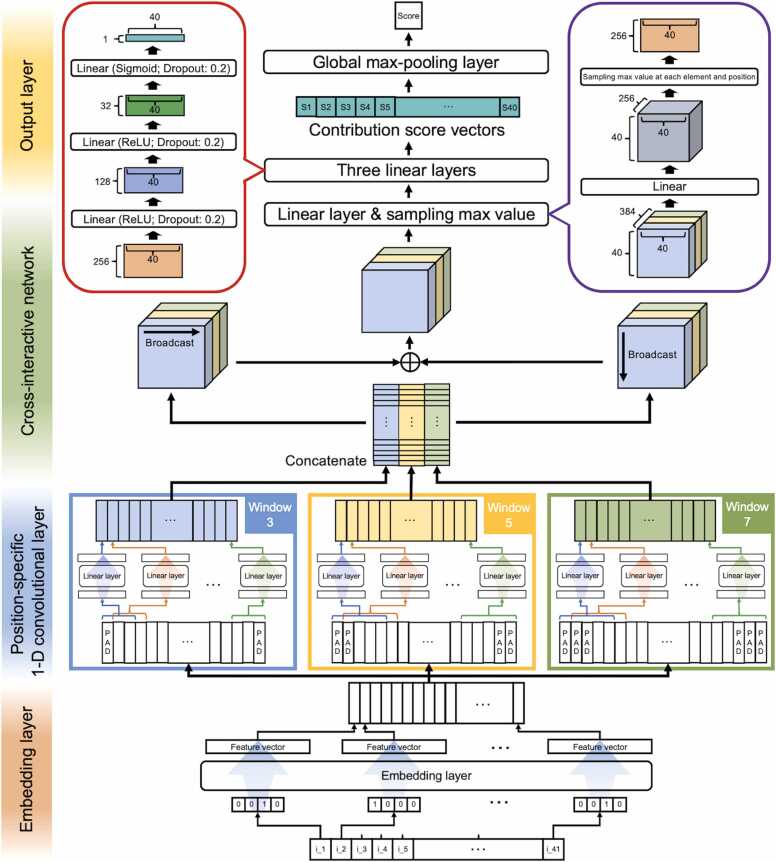
Network structure of CNN6mA. The CNN6mA is composed of four sub-networks. In the first sub-network (embedding layer), each nucleotide in the sequences is transformed to feature vectors. Then, the position-specific nucleotide patterns and their relationships are captured by the position-specific 1-D convolutional layers in the second sub-network and the cross-interactive network in the third sub-network, respectively. In the position-specific 1-D convolutional layers, “PAD” indicates zero-padding vectors to make the shapes of the output and input matrices the same. The position-specific 1-D convolutional layers use multi-scale filters with window sizes of 3, 5, and 7. In the final sub-network, the resulting feature tensor is transformed into the contribution score vectors by using the three fully connected layers to calculate the final score.

In this study, we have developed a CNN-based 6 mA site predictor, called CNN6mA. which proposed two new architectures: a position-specific 1-D convolutional layer and a cross-interactive network (Fig. 1). In the position-specific 1-D convolutional layer, position-specific filters with different window sizes were applied to an inquiry sequence instead of sharing the same filters over all positions in order to extract the position-specific features at different levels. The cross-interactive network explored the relationships between all the nucleotide patterns within the inquiry sequence. Consequently, CNN6mA outperformed the existing state-of-the-art models in many species and created the contribution score vector that intelligibly interpret the prediction mechanism.

## 2 Materials and methods

### 2.1 Dataset

To compare CNN6mA with existing state-of-the-art methods, we used a variety of the datasets that were retrieved from Lv et al.’s web application. [16], as shown in Table S1. The datasets were composed of 6 mA and non-6 mA sequences in 11 species including *A. thaliana*, *C. elegans*, *C. equisetifpolia*, *D. melanogaster*, *F. vesca*, *H. sapiens*, *R. chinensis*, *S. cerevisiae*, *T. thermophile*, *Ts. SUP5–1*, and *Xoc. BLS256*. We used the 41-bp DNA sequences with 6 mA and non-6 mA at the center as the positive and negative samples, respectively, and then removed the central adenine in both the samples, where the numbers of the two types of samples were the same in each dataset. The datasets were divided into the training and test data at a ratio of one to one.

### 2.2 Deep learning model

As shown in Fig. 1, our proposed deep learning model is composed of four sub-networks: the embedding layer, position-specific 1-D convolutional layer, cross-interactive network, and output layer. We proposed the two, novel networks of the position-specific 1-D convolutional layer and cross-interactive network. The position-specific 1-D convolutional layer uses different filters at each position to extract position-specific nucleotide information. In other words, the weights of the position-specific filters at each position are allowed to be changed or optimized in backpropagation. It differs from the normal 1-D convolutional layer that shares the same filters at all the positions. The cross-interactive network considers relationships between pairwise nucleotide patterns within an inquiry sequence.

In the embedding layer, nucleotides A, T, G, and C of the DNA sequence with a length of *N* are converted into indexes 1, 2, 3, and 4, respectively, and then each index is represented as an *M*-dimensional feature vector by nn.Embedding (PyTorch), where *M* set to 64. The resulting N×Mfeature matrix $X=[x_{i,j}]$ (1≤i≤N, 1≤j≤M) is inputted into the position-specific 1-D convolutional layer. We create *F* filters with window size *W* at each sequence position *i*, $\left[f_{i,k}{\left(W\right)}_{l,j}\right]$ (1≤k≤F), and apply them to $\left[x_{i,j}\right]$ generate output matrix $C=[c_{i,k}(W)]$, given by:

(1) $$c_{i,k}(W)=\sum_{l=1}^{W}\sum_{j=1}^{M}f_{i,k}{\left(W\right)}_{l,j}⊗x_{i-W/2+l-1,j},$$

where *F* is the number of filters and set to 128. For the convenience of explanation, the bias term is not displayed. During the training process, the weights of position-specific filters are optimized to capture position-specific sequence patterns. Zero-padding is applied to the resulting matrixes so that the shape of the input and output matrixes are the same. The output matrix is fed to the ReLU function and dropped out with a ratio of 0.2 to obtain the following matrix.

(2) $$d_{i,k}(W)=Dropout(ReLU(c_{i,k}(W))).$$

To capture feature patterns at different levels, we employ *F* filters with a different window size of $\left\{W_{1},W_{2},.,W_{S}\right\}$ and concatenate $d_{i,k}(W_{1})$, $d_{i,k}(W_{2})$,., $d_{i,k}(W_{S})$ at each position to obtain feature matrix B as follows:

(3) $$B=[b_{i,k}]=concat(d_{i,k}(W_{1}),d_{i,k}(W_{2}),.,d_{i,k}(W_{S})),$$

where *S* is the number of window sizes. The concatenated matrix is inputted into the cross-interactive network to generate feature tensor H that considers relationships between pairwise nucleotide patterns within an inquiry sequence. In the cross interactive network, matrix B is broadcasted in the two different axes. First, one dimension is added as the first dimension of B, and B is copied *N* times and expanded in the added axis, resulting in a tensor with a shape of N*×N×F, where * indicates the added dimension. Second, the other dimension is inserted at the second dimension of B, and B is copied *N* times and expanded in the added axis, resulting in a tensor with a shape of N×N*×F. The two resulting tensors are summed to obtain H, given by:

(4) $$H=[h_{i,p,k}]1\leq i,p\leq N,1\leq k\leq F.$$

After reducing the feature size of H by a fully connected layer to F′, we obtain feature matrix Q, given by:

(5) $$Q=[q_{i,k}]=\underset{1\leq p\leq N}{max}[h_{i,p,k}]1\leq k\leq F\prime$$

,where F′ set to 256. Then, Q is sent to the output layer composed of three fully connected layers, where the output feature size of the first, second, and third fully connected layers are set to 128, 32, and 1, respectively. Q is fed to the first and second fully connected layers with the ReLU function and dropout layer (with a ratio of 0.2), and then fed to the third fully connected layer with the sigmoid function to produce the final output vector whose elements are values in a range of 0–1. The final output vector is defined as the contribution score vector that provides interpretability of the prediction. The final score is obtained by sampling a max value element from the final output vector.

Applying the global max-pooling layer to the last layer provided the contribution vector elements corresponding to the 6 mA-related nucleotide positions with a value close to 1. For example, if nucleotide patterns co-occurring with 6 mA exist, the global max-pooling layer outputs a higher value at those positions. It is because the neural networks are optimized so that the global max-pooling layer can give a value close to 1 to the positive samples. In other words, the mechanism for identifying the positions significantly responsible for positive samples is incorporated. CNN6mA presents the nucleotide positions that contributed to the 6 mA prediction through this contribution score vector to explain the prediction. The construction of the deep learning model is executed by PyTorch of the Python package [29].

### 2.3 Training and evaluation

We trained and validated the neural network model via 5-fold cross-validation with training data. Mini-batch learning was applied for the training with a batch size of 64. The optimization was executed by the Adam optimizer with a learning rate of $1.0\times 10^{-5}$ and was stopped when the maximum value of AUC in the validation data was not updated for consecutive 30 epochs.

The trained models were evaluated by the independent test with test data. For the evaluation, 5 statistical measures were adopted: sensitivity (SN; recall), specificity (SP), accuracy (ACC), and Matthews correlation coefficient (MCC) provided by:

(6) $$\begin{array}{c} \mathrm{SN}=\frac{\mathrm{TP}}{\mathrm{TP}+\mathrm{FN}} \end{array}$$

(7) $$\begin{array}{c} \mathrm{SP}=\frac{\mathrm{TN}}{\mathrm{TN}+\mathrm{FP}} \end{array}$$

(8) $$\begin{array}{c} \mathrm{ACC}=\frac{\mathrm{TP}+\mathrm{TN}}{\mathrm{TP}+\mathrm{TN}+\mathrm{FP}+\mathrm{FN}} \end{array}$$

(9) $$\begin{array}{c} \mathrm{MCC}=\frac{\mathrm{TP}\times \mathrm{TN}-\mathrm{FP}\times \mathrm{FN}}{\sqrt{\left(\mathrm{TN}+\mathrm{FN}\right)\times \left(\mathrm{TP}+\mathrm{FP}\right)\times \left(\mathrm{TN}+\mathrm{FP}\right)\times \left(\mathrm{TP}+\mathrm{FN}\right)}} \end{array}$$

where *TP*, *FP*, *TN*, and *FN* indicate the numbers of true-positive, false-positive, true-negative, and false-negative samples, respectively. AUC is the area under the receiver operating characteristic curve given by plotting the *SN* with respect (1-*SP*) at various threshold settings. The threshold for separating positive samples from negative samples was set to 0.5. The statistical measures of 5 models were averaged. The calculation of the measurements was executed by scikit-learn [30] of the Python package.

### 2.4 Analysis of contribution score vectors

Before analysis, the contribution score vectors were smoothed by averaging n consecutive score elements. Three smoothed contribution scores in n of 3, 5, and 7 were generated and averaged at each position. To observe the differences between contribution score vectors in positive and negative samples, we visualized the contribution score vectors by two methods for dimensional reduction: t-distributed stochastic neighbor embedding (t-SNE) [31] and uniform manifold approximation and projection (UMAP) [32]. In the t-SNE, the low dimensional data was generated by bringing the distribution of distances between the actual data points closer to that in low dimensional space. Perplexity which determines the variance in the distribution was set to 50. On the other hand, in the UMAP, data points in low dimensional space are determined by working repulsion and attraction between data points in a k-nearest neighbor graph. The number of neighbors in the k-neighbor graph and minimum distances between points were set to 30 and 0.15, respectively.

Furthermore, we explored the nucleotide patterns which present a higher value at a specific position in the contribution score vector. Concretely, we extracted the samples whose contribution scores satisfy the two following conditions at the target position:

(10) $$c_{\mathrm{target}}>\mu +k\sigma$$

(11) $$c_{\mathrm{target}}>0.5,$$

where $c_{\mathrm{target}}$, μ, σ, and k indicate the contribution score at the target position, the mean and standard deviation of the contribution score over all positions, and a constant. k was set to 2. We focused on the positions at which the number of the samples satisfying the conditions was more than 50.

## 3 Results and discussion

### 3.1 Optimization of CNN6mA

**Fig. 2:**
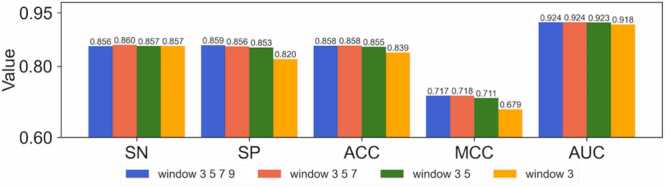
Effect of the combination of multi-scale filters on the performance of CNN6mA in 5-fold cross-validation. The 1-D convolutional layer employs a filter with a window size of 3, two filters with window sizes of 3 and 5, three filters with window sizes of 3, 5, and 7, and four filters with window sizes of 3, 5, 7, and 9.

To extract position-specific nucleotide patterns, the position-specific filters were created in the 1-D convolutional layers of CNN6mA. In addition to the position-specific feature extraction, to further capture various nucleotide patterns, the multi-scale feature extraction was conducted by using filters with different window sizes of 3, 5, 7, and 9. In previous studies in DNA-protein binding site prediction, the use of multi-scale filters improved the performances [33], [34]. We investigated the optimal combinations of multi-scale filters in position-specific feature extraction. Concretely, we constructed a filter with a window size of 3, two filters with window sizes of 3 and 5, three filters with window sizes of 3, 5, and 7, and four filters with window sizes of 3, 5, 7, and 9. We trained the CNN6mA model via 5-fold cross-validation with the respective training dataset of 11 species, where the statistical measures of the resulting 5 models in all species were averaged. As shown in Fig. 2, the combination of three filters with window sizes of 3, 5, and 7 achieved the highest SN, ACC, MCC, and AUC, indicating that the combination of multi-scale filters is effective in enhanced prediction. Theoretically, a long-size filter extracts information of long sequence patterns; a short-size filter is not able to capture the information of such a long motif all at once. A window size of 9 might be too long to capture nucleotide patterns. Finally, we implemented three position-specific filters with window sizes of 3, 5, and 7 in the 1-D convolutional layers of CNN6mA.

### 3.2 Comparison of CNN6mA with existing state-of-the-art methods

**Fig. 3:**
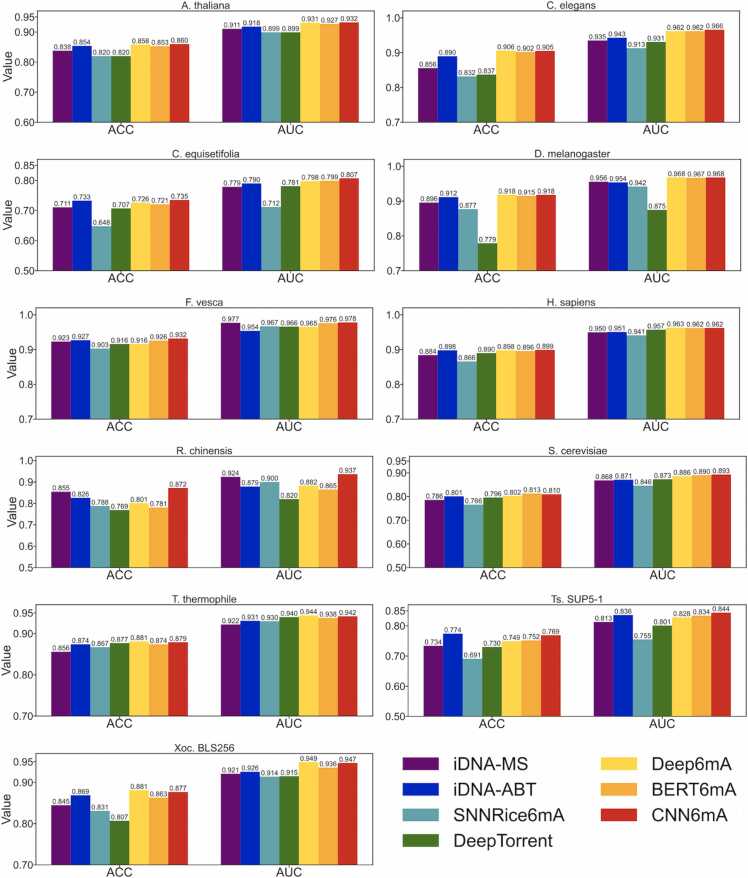
Performance comparison of CNN6mA with the state-of-the-art methods in the independent tests for 11 species. The performances of iDNA-MS, iDNA-ABT, SNNRice6mA, DeepTorrent, and BERT6mA are given from Table S3 in Lv et al.’s paper (iDNA-MS), Table S2 in Yu et al.’s paper (iDNA-ABT), and Tables S2-S12 in our previous paper (BERT6mA). AUCs in Deep6mA are given from Fig. 4 in our previous paper (BERT6mA) and ACCs are calculated with the BERT6mA-trained models.

We compared the performances of CNN6mA with those of existing state-of-the-art models including iDNA-MS, iDNA-ABT, SNNRice6mA, DeepTorrent, Deep6mA, and BERT6mA. The performances of iDNA-MS, iDNA-ABT, SNNRice6mA, DeepTorrent, and BERT6mA were evaluated in our previous paper [26]. We trained Deep6mA via 5-fold cross-validation with the training data and evaluated them with the independent test data. As shown in Fig. 3 and Table S2, CNN6mA presented the highest ACCs and AUCs in 6 and 8 species out of the 11 species, respectively, suggesting that CNN6mA has a high learning ability and generalizability. We consider that the high learning ability of CNN6mA is attributed to the position-specific filters of the 1-D convolutional layers and the cross-interactive network, which capture the position-associated information and the relationships between local nucleotide patterns, respectively. The usefulness of those two architectures will be demonstrated later.

The iDNA-MS presented high performances on the small datasets of *F. vesca* and *R. chinensis*, showing the effectiveness of machine learning on small datasets, respectively. CNN6mA presented high performances on small datasets, but the other deep learning-based models did not, suggesting the learning potential of CNN6mA on various data sizes. The BERT6mA, iDNA-ABT, and Deep6mA presented comparable performances to the CNN6mA in many species. The first two models could extract the context information based on the BERT-based neural networks; the Deep6mA model could capture some nucleotide patterns based on LSTM and CNN-based neural networks.

### 3.3 Effectiveness of position-specific 1-D convolutional layer

**Fig. 4:**
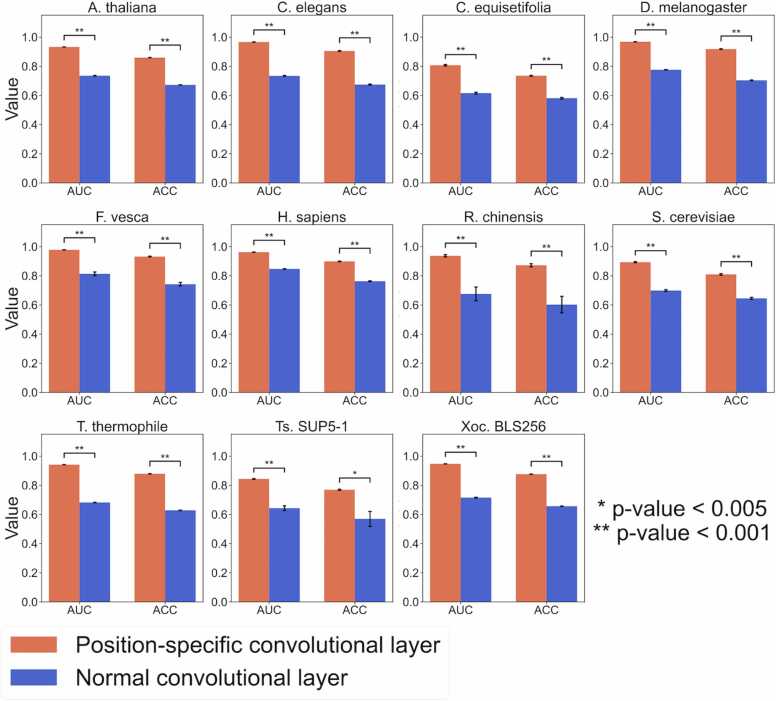
Performance comparison of CNN6mA with the position-specific 1-D convolutional layer with that with the normal 1-D convolutional layer. The index-embedding method is used. One-sided paired-sample t-test is used.

To investigate the effectiveness of the position-specific filters in the 1-D convolutional layer, we compared the performances of the multi-scale, position-specific 1-D convolutional layer that use the position-specific filters with those of the normal multi-scale 1-D convolutional layer that shares the same filters at all positions. We used the two different encoding methods: the index embedding method (see Materials and methods) and one-hot encoding. The one-hot encoding converts A, T, G, and C into (1,0,0,0), (0,1,0,0) (0,0,1,0), and (0,0,0,1), respectively. As shown in Fig. 4 and S1, and Tables S3-S6, use of the position-specific 1-D convolutional layer presented significantly higher ACCs and AUCs than use of the normal 1-D convolutional layer in almost all species in both the encoding methods (one-sided paired-sample t-test), suggesting that use of the position-specific filters is effective in enhanced prediction. This explicitly indicates the usefulness of the position-specific information for 6 mA prediction.

### 3.4 Effect of cross-interactive network

**Fig. 5:**
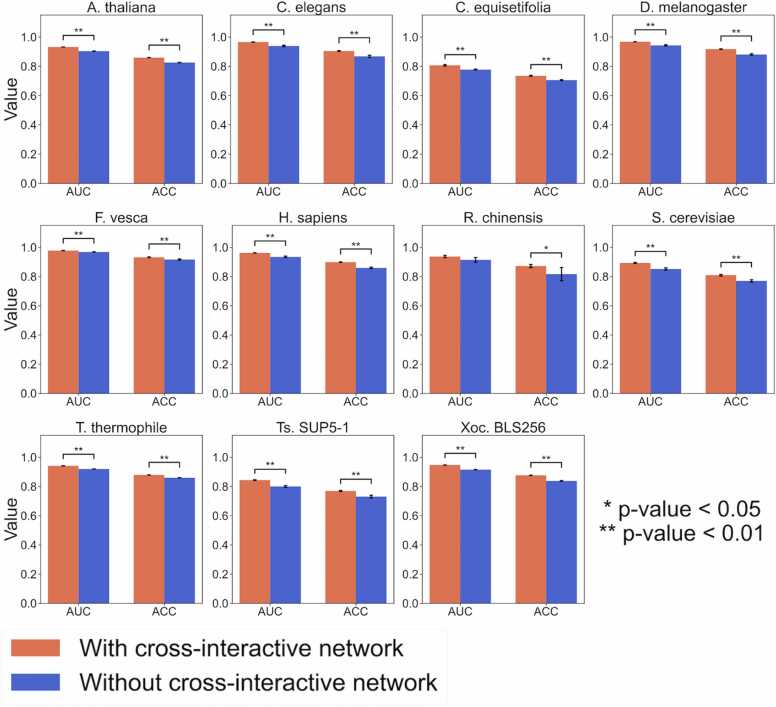
Effect of the cross-interactive network on the prediction performance of CNN6mA in independent tests.

While the proposed position-specific 1-D convolutional layer captures nucleotide patterns at specific positions, it is not able to extract the relationships between those patterns at a distance. To complement this issue and represent the relationships between nucleotide patterns within an inquiry sequence, we created the cross-interactive network. This network superimposes the features between all the nucleotide pairs in the inquiry sequence to explore some relationships between them. To validate the usefulness of the cross-interactive network-based feature extraction, we compared the CNN6mA including the cross-interactive network with the CNN6mA without it (Fig. S2). Notably, the performance increased while keeping the numbers of parameters in the two types of models the same. As shown in Fig. 5 and Tables S3 and S7, use of the cross-interactive network significantly increased the prediction performance (one-sided paired-sample t-test), demonstrating that the cross-interactive network is useful for the prediction of 6 mA sites.

### 3.5 Interpretation of prediction mechanism

**Fig. 6:**
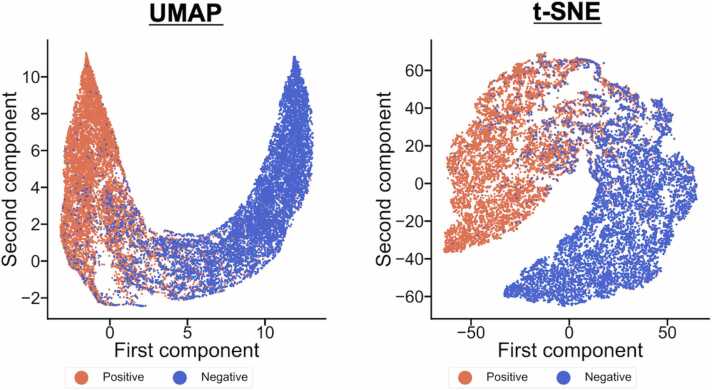
Visualization of the contribution score vectors for the negative and positive samples by UMAP (left) and (t-SNE).

**Fig. 7:**
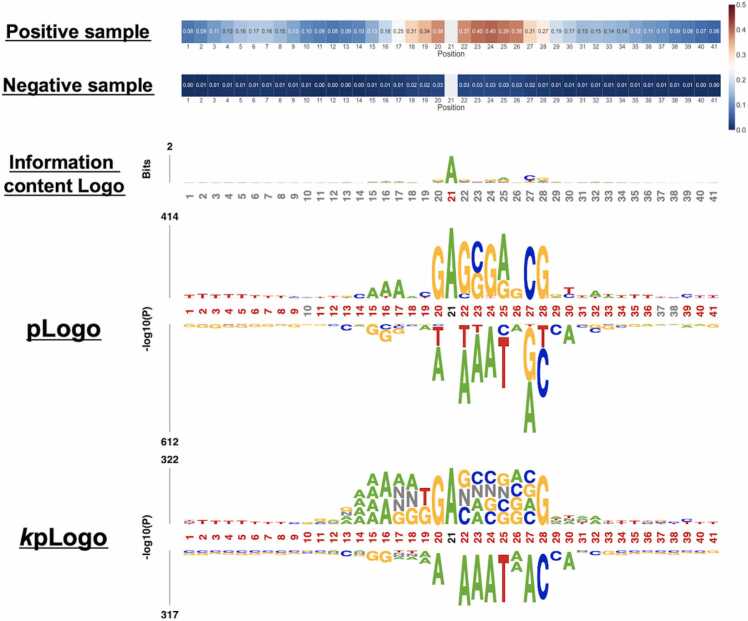
Heat map of the contribution score vector. In the positive (top) and negative samples (bottom), the contribution score vectors generated by the independent test of *H. sapiens* are averaged (Upper panel). Nucleotide preference profiles in the test data of *H. sapiens*. They are visualized by three tools of the information content Logo (top), pLogo (middle), and *k*pLogo (bottom) (Lower panel).

CNN6mA proposed the contribution score vector for the interpretation of prediction mechanisms. To exemplify the contribution score vectors, we visualized them in the independent test in *H. sapiens* by t-SNE and UMAP. The averaged contribution score vectors of the positive and negative samples were separated clearly (Fig. 6), indicating that they are critically responsible for classification. Those in the positive samples have higher values than those in negative samples (Fig. 7). In the positive samples, we observed that the positions with a high score agreed to those at which the preferred nucleotides appeared on the sequence logos, resulted from the information content Logo [35], pLogo [36], and *k*pLogo [35] (Fig. 7). Specifically, the high-value elements in the vector and the preferred nucleotides on the sequence logos were observed at middle positions from 18 to 28.

**Fig. 8:**
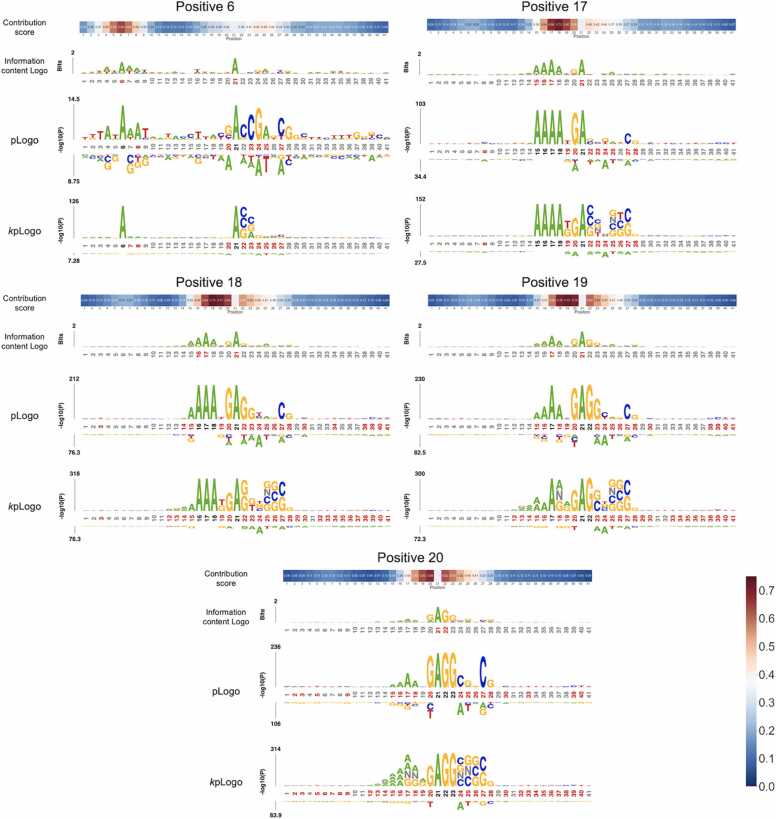
Contribution score vectors and nucleotide patterns with a high score at the upstream positions of 6 mA. At positions of 6, 17, 18, 19, and 20, many samples have a high score at specific positions. The preferred nucleotides are visualized by three tools of the information content Logo, pLogo, and *k*pLogo.

**Fig. 9:**
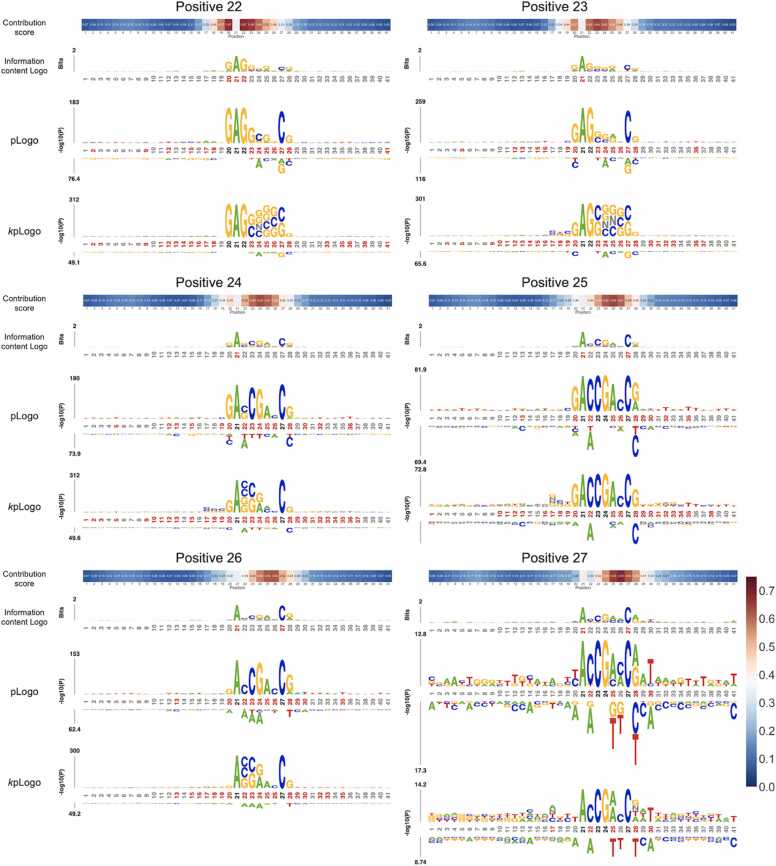
Contribution score vectors and nucleotide patterns with a high score at the upstream positions of 6 mA. At positions of 22, 23, 24, 25, 26, and 27, many samples have a high score at specific positions. The preferred nucleotides are visualized by three tools of the information content Logo, pLogo, and *k*pLogo.

To investigate the interpretability of the contribution score vectors, we collected the samples with a high score in the contribution vector and visualized those samples by the sequence logo (see Materials and methods). We focused on the positions where the number of samples with a high score is more than 50 and averaged those score vectors. As shown in Fig. 8, Fig. 9, the preferred nucleotide patterns appeared around the position with a high score in the contribution score vectors. For example, the sequences with a high score at position 20 contained a GAGG motif which was involved in actively transcribed genes in previous study [9]. In addition, the sequences with high scores at positions 17 and 24 included the AAAA motif and CG motif, in the same manner as the sequence logs. These results suggest that the contribution score vectors reflect the positions of preferred nucleotides in the 6 mA samples.

DNA motifs are generally related to DNA binding proteins and non-coding RNAs during DNA methylation processes. Previous studies investigated the relationship between the motifs and DNA methylation/demethylation and emphasized the importance of the motifs for establishing and maintaining the methylation [37], [38], [39], [40] in addition to the locus-specificity of methylation [37]. CNN6mA created the contribution score vectors that reflect the 6 mA-associated nucleotide patterns and can be useful for finding motifs. While motif exploration tools such as sequence Logo provide the comprehensive result over a sufficient number of samples, the contribution score vectors explicitly tell the critical positions and 6 mA-associated nucleotide patterns for each sample sequence. For example, as shown in Fig. 7, the pLogo could not identify any preferential nucleotides in the upstream positions from 1 to 10 for all samples, while it could find adenine at position 6 only for the samples with a high contribution score. This is a great advantage of the contribution vector over the motif exploration tools.

BERT6mA [26] interpreted the prediction mechanism through attention weights that indicate the relationships between *k*-mer sequences. Since the attention weight was generated for each *k*-mer, not for each nucleotide, it was difficult to specify the 6 mA-related nucleotides. Compared to such attention weights, the CNN6mA-generated contribution score vectors, which are calculated based on the information of specific 6 mA-related nucleotides and their relationships, explicitly evaluate a contribution degree at each nucleotide. This would make it more intuitive to find 6 mA-related patterns than the attention weight.

### 3.6 Web server implementation

To promote research in the field of epigenetics and genome analysis, a web server application of CNN6mA was constructed and can be freely accessed from http://kurata35.bio.kyutech.ac.jp/CNN6mA/. Flask (1.1.2) in a python package (3.8.0) and apache (2.4.18) were used for implementation. The application requires DNA sequences with a length of 41 bp and an adenine positioned at the center in the FASTA format and outputs predictive scores and the contribution score vectors. The details of the application are provided on the help page of the site.

## 4 Conclusion

We constructed an interpretable CNN-based model for DNA 6 mA site prediction, named CNN6mA. In the present study, we suggest three novel neural network architectures: position-specific 1D convolutional layer, a cross-interactive network, and a global max-pooling layer-based network to generate the contribution score vectors. We showed that the first two architectures improved the prediction performances. Based on those architectures, in the benchmark, CNN6mA outperformed the state-of-the-art methods in many species. The third architecture generates the contribution score vectors that exhibit the neural network-focused positions. The contribution score vectors could be useful for finding 6 mA-associated motifs with a small number of samples. In the near future, we will find the species-common and species-specific motif patterns by using the contribution scores and examine their motif patterns and biological meanings to understand new epigenetic mechanisms.

## CRediT authorship contribution statement

**Sho Tsukiyama**: Conceptualization, Methodology, Software, Formal analysis, Writing – original draft, and Writing – review & editing. **Md Mehedi Hasan**: Writing – original draft, Writing – review & editing. **Hiroyuki Kurata**: Conceptualization, Methodology, Writing – original draft, and Writing – review & editing.

## Declaration of Competing Interest

All authors declare that they have no conflicts of interest.

## Data Availability

The source codes of CNN6mA are freely accessible at https://github.com/kuratahiroyuki/CNN6mA.git, respectively.
