# Supplementary material for: CNN6mA: Interpretable neural network model based on position-specific CNN and cross-interactive network for 6mA site prediction
Source: Comput Struct Biotechnol J. 2022 Dec 28;21:644–54. doi: 10.1016/j.csbj.2022.12.043 (PMC9826936; doi:10.1016/j.csbj.2022.12.043)
Supplement: Supplementary file 1 — Supplementary material [file mmc1.docx]

# Supplemental material


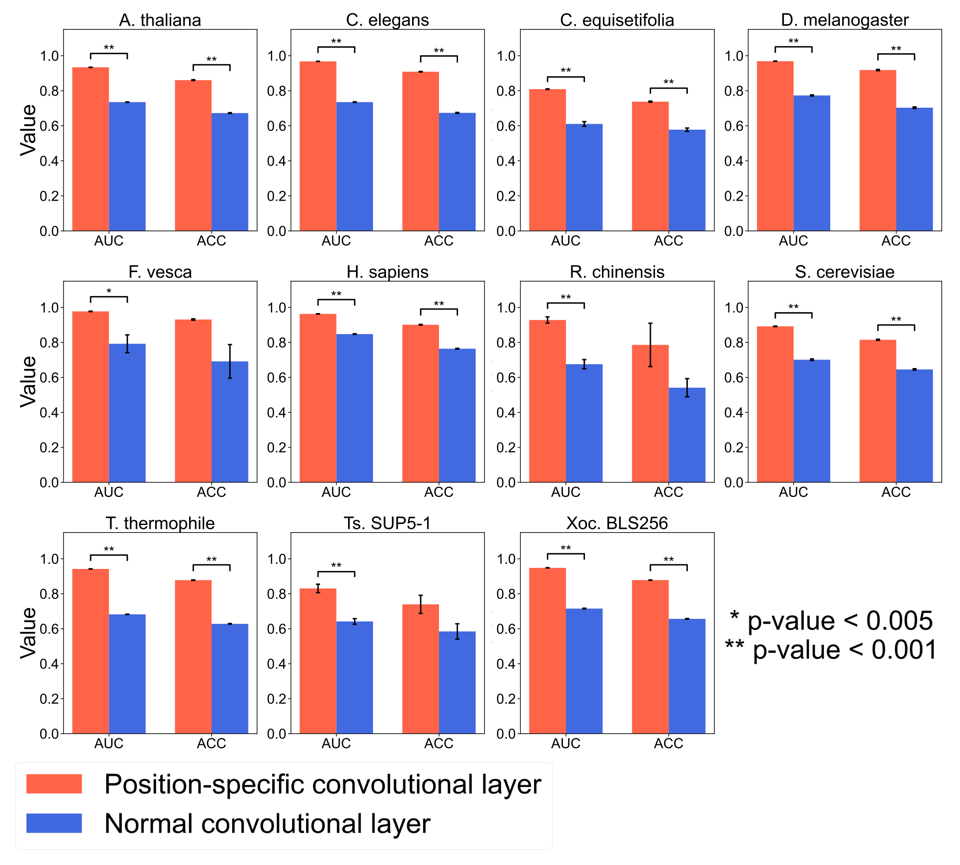


**Fig. S1.** Performance comparison of CNN6mA with the position-specific 1-D convolutional layer with that with the normal 1-D convolutional layer. The one-hot binary encoding method is used. One-sided paired-sample t-test is used.


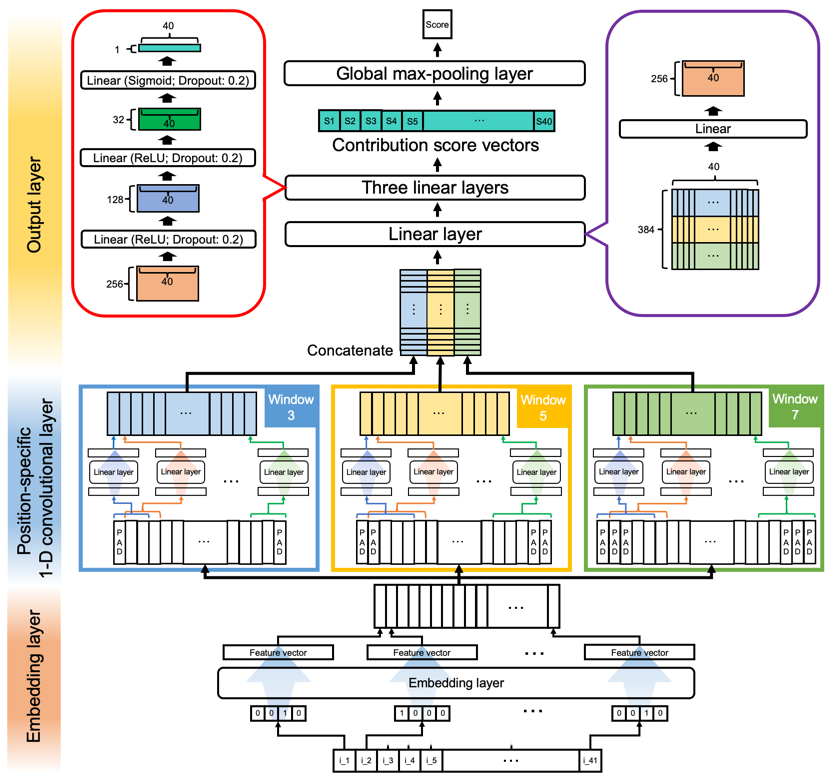


**Fig. S2.** Network structure of CNN6mA without the cross-interactive network. After concatenating the output vectors from the position-specific 1-D convolutional layer, the resulting matrixes are sent to the output layer.

**Table S1.** Statistical features of the Lv et al.’s 6mA datasets

| **Species** | **Training data** | | **Test data** | |
| --- | --- | --- | --- | --- |
|  | **Positive sample** | **Negative sample** | **Positive sample** | **Negative sample** |
| *A. thaliana* | 15937 | 15937 | 15936 | 15936 |
| *C. elegans* | 3981 | 3981 | 3980 | 3980 |
| *C. equisetifolia* | 3033 | 3033 | 3033 | 3033 |
| *D. melanogaster* | 5596 | 5596 | 5595 | 5595 |
| *F. vesca* | 1551 | 1551 | 1551 | 1551 |
| *H. sapiens* | 9168 | 9168 | 9167 | 9167 |
| *R. chinensis* | 300 | 300 | 299 | 299 |
| *S. cerevisiae* | 1893 | 1893 | 1893 | 1893 |
| *T. thermophile* | 53800 | 53800 | 53800 | 53800 |
| *Ts. SUP5-1* | 1690 | 1690 | 1689 | 1689 |
| *Xoc. BLS256* | 8608 | 8608 | 8607 | 8607 |

**Table. S2.** Comparison of CNN6mA with the-state-of-the-art methods in the independent tests of 11 species. The performances in iDNA-MS, iDNA-ABT, SNNRice6mA, DeepTorrent, and BERT6mA are given from the Table S3 in Lv et al.’s paper (iDNA-MS), Table S2 in Yu et al’s paper (iDNA-ABT), and Tables S2-S12 in our previous paper (BERT6mA). AUCs in Deep6mA are given from Fig. 4 in our previous paper (BERT6mA), and the other measurements are calculated with the BERT6mA-trained models.

| **Species** | **Model** | **SN** | **SP** | **ACC** | **MCC** | **AUC** |
| --- | --- | --- | --- | --- | --- | --- |
| *A. thaliana* | iDNA-MS | 0.824 | 0.851 | 0.838 | 0.676 | 0.911 |
|  | iDNA_ABT | 0.823 | 0.884 | 0.854 | 0.709 | 0.918 |
|  | SNNRice6mA | 0.793 | 0.846 | 0.820 | 0.641 | 0.899 |
|  | DeepTorrent | 0.767 | 0.872 | 0.820 | 0.643 | 0.899 |
|  | Deep6mA | 0.823 | **0.894** | 0.858 | 0.719 | 0.931 |
|  | BERT6mA | **0.846** | 0.859 | 0.853 | 0.705 | 0.927 |
|  | CNN6mA | 0.846 | 0.873 | **0.860** | **0.719** | **0.932** |
| *C. elegans* | iDNA-MS | 0.868 | 0.844 | 0.856 | 0.712 | 0.935 |
|  | iDNA_ABT | 0.882 | 0.899 | 0.890 | 0.781 | 0.943 |
|  | SNNRice6mA | 0.872 | 0.792 | 0.832 | 0.666 | 0.913 |
|  | DeepTorrent | 0.728 | **0.946** | 0.837 | 0.691 | 0.931 |
|  | Deep6mA | 0.913 | 0.898 | **0.906** | **0.812** | 0.962 |
|  | BERT6mA | 0.908 | 0.895 | 0.902 | 0.803 | 0.962 |
|  | CNN6mA | **0.924** | 0.886 | 0.905 | 0.811 | **0.966** |
| *C. equisetifolia* | iDNA-MS | 0.718 | 0.705 | 0.711 | 0.423 | 0.779 |
|  | iDNA_ABT | 0.689 | 0.777 | 0.733 | 0.467 | 0.790 |
|  | SNNRice6mA | 0.645 | 0.651 | 0.648 | 0.297 | 0.712 |
|  | DeepTorrent | 0.590 | **0.823** | 0.707 | 0.425 | 0.781 |
|  | Deep6mA | **0.728** | 0.723 | 0.726 | 0.452 | 0.798 |
|  | BERT6mA | 0.707 | 0.736 | 0.721 | 0.443 | 0.799 |
|  | CNN6mA | 0.696 | 0.774 | **0.735** | **0.472** | **0.807** |
| *D. melanogaster* | iDNA-MS | 0.890 | 0.903 | 0.896 | 0.792 | 0.956 |
|  | iDNA_ABT | 0.904 | 0.921 | 0.912 | 0.824 | 0.954 |
|  | SNNRice6mA | 0.871 | 0.883 | 0.877 | 0.754 | 0.942 |
|  | DeepTorrent | 0.630 | **0.927** | 0.779 | 0.584 | 0.875 |
|  | Deep6mA | **0.922** | 0.913 | 0.918 | 0.835 | 0.968 |
|  | BERT6mA | 0.913 | 0.917 | 0.915 | 0.830 | 0.967 |
|  | CNN6mA | 0.915 | 0.921 | **0.918** | **0.836** | **0.968** |
| *F. vesca* | iDNA-MS | **0.939** | 0.906 | 0.923 | 0.846 | 0.977 |
|  | iDNA_ABT | 0.923 | **0.930** | 0.927 | 0.824 | 0.954 |
|  | SNNRice6mA | 0.914 | 0.892 | 0.903 | 0.807 | 0.967 |
|  | DeepTorrent | 0.903 | 0.928 | 0.916 | 0.832 | 0.966 |
|  | Deep6mA | 0.920 | 0.912 | 0.916 | 0.832 | 0.965 |
|  | BERT6mA | 0.925 | 0.926 | 0.926 | 0.851 | 0.976 |
|  | CNN6mA | 0.938 | 0.926 | **0.932** | **0.864** | **0.978** |
| *H. sapiens* | iDNA-MS | 0.863 | 0.905 | 0.884 | 0.769 | 0.950 |
|  | iDNA_ABT | 0.894 | 0.902 | 0.898 | 0.796 | 0.951 |
|  | SNNRice6mA | 0.862 | 0.871 | 0.866 | 0.733 | 0.941 |
|  | DeepTorrent | 0.869 | **0.911** | 0.890 | 0.780 | 0.957 |
|  | Deep6mA | **0.908** | 0.888 | 0.898 | 0.797 | **0.963** |
|  | BERT6mA | 0.891 | 0.901 | 0.896 | 0.792 | 0.962 |
|  | CNN6mA | 0.896 | 0.902 | **0.899** | **0.798** | 0.962 |
| *R. chinensis* | iDNA-MS | 0.880 | 0.829 | 0.855 | 0.710 | 0.924 |
|  | iDNA-ABT | 0.809 | 0.843 | 0.826 | 0.653 | 0.879 |
|  | SNNRice6mA | 0.772 | 0.804 | 0.788 | 0.577 | 0.900 |
|  | DeepTorrent | 0.806 | 0.732 | 0.769 | 0.540 | 0.820 |
|  | Deep6mA | 0.769 | 0.833 | 0.801 | 0.603 | 0.882 |
|  | BERT6mA | 0.743 | 0.819 | 0.781 | 0.564 | 0.865 |
|  | CNN6mA | **0.898** | **0.847** | **0.872** | **0.746** | **0.937** |
| *S. cerevisiae* | iDNA-MS | 0.754 | 0.817 | 0.786 | 0.572 | 0.868 |
|  | iDNA_ABT | 0.724 | **0.879** | 0.801 | 0.610 | 0.871 |
|  | SNNRice6mA | 0.762 | 0.771 | 0.766 | 0.533 | 0.846 |
|  | DeepTorrent | 0.764 | 0.829 | 0.796 | 0.594 | 0.873 |
|  | Deep6mA | 0.745 | 0.859 | 0.802 | 0.608 | 0.886 |
|  | BERT6mA | **0.801** | 0.825 | **0.813** | **0.627** | 0.890 |
|  | CNN6mA | 0.795 | 0.824 | 0.810 | 0.620 | **0.893** |
| *T. thermophile* | iDNA-MS | **0.958** | 0.755 | 0.856 | 0.728 | 0.922 |
|  | iDNA_ABT | 0.933 | 0.815 | 0.874 | 0.754 | 0.931 |
|  | SNNRice6mA | 0.940 | 0.793 | 0.867 | 0.741 | 0.930 |
|  | DeepTorrent | 0.939 | 0.816 | 0.877 | 0.760 | 0.940 |
|  | Deep6mA | 0.941 | 0.821 | **0.881** | **0.768** | **0.944** |
|  | BERT6mA | 0.925 | **0.823** | 0.874 | 0.752 | 0.938 |
|  | CNN6mA | 0.938 | 0.820 | 0.879 | 0.763 | 0.942 |
| *Ts. SUP5-1* | iDNA-MS | 0.743 | 0.726 | 0.734 | 0.468 | 0.813 |
|  | iDNA_ABT | 0.718 | 0.830 | **0.774** | **0.551** | 0.836 |
|  | SNNRice6mA | 0.665 | 0.716 | 0.691 | 0.382 | 0.755 |
|  | DeepTorrent | 0.623 | **0.838** | 0.730 | 0.472 | 0.801 |
|  | Deep6mA | 0.753 | 0.745 | 0.749 | 0.498 | 0.828 |
|  | BERT6mA | **0.772** | 0.732 | 0.752 | 0.505 | 0.834 |
|  | CNN6mA | 0.749 | 0.790 | 0.769 | 0.539 | **0.844** |
| *Xoc. BLS256* | iDNA-MS | 0.825 | 0.865 | 0.845 | 0.691 | 0.921 |
|  | iDNA_ABT | 0.889 | 0.849 | 0.869 | 0.739 | 0.926 |
|  | SNNRice6mA | 0.850 | 0.812 | 0.831 | 0.663 | 0.914 |
|  | DeepTorrent | **0.941** | 0.667 | 0.807 | 0.634 | 0.915 |
|  | Deep6mA | 0.876 | **0.885** | **0.881** | **0.761** | **0.949** |
|  | BERT6mA | 0.848 | 0.878 | 0.863 | 0.726 | 0.936 |
|  | CNN6mA | 0.880 | 0.873 | 0.877 | 0.754 | 0.947 |

**Table. S3.** Performances of CNN6mA with the position-specific 1-D convolutional layer in independent test. The index embedding method was used.

| **Species** | **SN** | **SP** | **ACC** | **MCC** | **AUC** |
| --- | --- | --- | --- | --- | --- |
| *A.thaliana* | 0.846 | 0.873 | 0.860 | 0.719 | 0.932 |
| *C.elegans* | 0.924 | 0.886 | 0.905 | 0.811 | 0.966 |
| *C.equisetifolia* | 0.696 | 0.774 | 0.735 | 0.472 | 0.807 |
| *D.melanogaster* | 0.915 | 0.921 | 0.918 | 0.836 | 0.968 |
| *F.vesca* | 0.938 | 0.926 | 0.932 | 0.864 | 0.978 |
| *H.sapiens* | 0.896 | 0.902 | 0.899 | 0.798 | 0.962 |
| *R.chinensis* | 0.898 | 0.847 | 0.872 | 0.746 | 0.937 |
| *S.cerevisiae* | 0.795 | 0.824 | 0.810 | 0.620 | 0.893 |
| *T.thermophile* | 0.938 | 0.820 | 0.879 | 0.763 | 0.942 |
| *Ts. SUP5-1* | 0.749 | 0.790 | 0.769 | 0.539 | 0.844 |
| *Xoc. BLS256* | 0.880 | 0.873 | 0.877 | 0.754 | 0.947 |

**Table. S4.** Performances of CNN6mA with the 1-D normal convolutional layer in independent test. The index embedding method was used.

| **Species** | **SN** | **SP** | **ACC** | **MCC** | **AUC** |
| --- | --- | --- | --- | --- | --- |
| *A.thaliana* | 0.631 | 0.714 | 0.672 | 0.347 | 0.735 |
| *C.elegans* | 0.663 | 0.686 | 0.674 | 0.349 | 0.734 |
| *C.equisetifolia* | 0.589 | 0.573 | 0.581 | 0.163 | 0.616 |
| *D.melanogaster* | 0.683 | 0.724 | 0.703 | 0.409 | 0.776 |
| *F.vesca* | 0.743 | 0.741 | 0.742 | 0.484 | 0.814 |
| *H.sapiens* | 0.741 | 0.785 | 0.763 | 0.527 | 0.847 |
| *R.chinensis* | 0.484 | 0.720 | 0.602 | 0.223 | 0.675 |
| *S.cerevisiae* | 0.620 | 0.671 | 0.645 | 0.291 | 0.699 |
| *T.thermophile* | 0.587 | 0.669 | 0.628 | 0.258 | 0.682 |
| *Ts. SUP5-1* | 0.382 | 0.757 | 0.570 | 0.159 | 0.643 |
| *Xoc. BLS256* | 0.600 | 0.714 | 0.657 | 0.316 | 0.716 |

**Table. S5.** Performances of CNN6mA with the position-specific 1-D convolutional layer in independent test. The one-hot encoding method was used.

| **Species** | **SN** | **SP** | **ACC** | **MCC** | **AUC** |
| --- | --- | --- | --- | --- | --- |
| *A.thaliana* | 0.854 | 0.867 | 0.861 | 0.722 | 0.934 |
| *C.elegans* | 0.925 | 0.892 | 0.909 | 0.818 | 0.968 |
| *C.equisetifolia* | 0.707 | 0.768 | 0.738 | 0.477 | 0.809 |
| *D.melanogaster* | 0.927 | 0.909 | 0.918 | 0.837 | 0.969 |
| *F.vesca* | 0.944 | 0.919 | 0.931 | 0.862 | 0.978 |
| *H.sapiens* | 0.887 | 0.915 | 0.901 | 0.803 | 0.963 |
| *R.chinensis* | 0.930 | 0.642 | 0.786 | 0.609 | 0.928 |
| *S.cerevisiae* | 0.783 | 0.847 | 0.815 | 0.632 | 0.892 |
| *T.thermophile* | 0.923 | 0.832 | 0.878 | 0.759 | 0.942 |
| *Ts. SUP5-1* | 0.664 | 0.815 | 0.739 | 0.496 | 0.830 |
| *Xoc. BLS256* | 0.873 | 0.883 | 0.878 | 0.757 | 0.948 |

**Table. S6.** Performances of CNN6mA with the 1-D normal convolutional layer in independent test. The one-hot encoding method was used.

| **Species** | **SN** | **SP** | **ACC** | **MCC** | **AUC** |
| --- | --- | --- | --- | --- | --- |
| *A.thaliana* | 0.664 | 0.681 | 0.673 | 0.346 | 0.735 |
| *C.elegans* | 0.673 | 0.675 | 0.674 | 0.348 | 0.735 |
| *C.equisetifolia* | 0.521 | 0.635 | 0.578 | 0.157 | 0.611 |
| *D.melanogaster* | 0.699 | 0.707 | 0.703 | 0.406 | 0.773 |
| *F.vesca* | 0.560 | 0.823 | 0.692 | 0.385 | 0.792 |
| *H.sapiens* | 0.734 | 0.795 | 0.764 | 0.530 | 0.848 |
| *R.chinensis* | 0.688 | 0.394 | 0.541 | 0.091 | 0.675 |
| *S.cerevisiae* | 0.614 | 0.677 | 0.646 | 0.292 | 0.701 |
| *T.thermophile* | 0.578 | 0.678 | 0.628 | 0.258 | 0.682 |
| *Ts. SUP5-1* | 0.461 | 0.708 | 0.584 | 0.173 | 0.642 |
| *Xoc. BLS256* | 0.579 | 0.734 | 0.656 | 0.317 | 0.715 |

**Table. S7.** Performances of the CNN6mA without the cross-interactive network in independent test. The index embedding method and the position-specific 1-D convolutional layer were used.

| **Species** | **SN** | **SP** | **ACC** | **MCC** | **AUC** |
| --- | --- | --- | --- | --- | --- |
| *A.thaliana* | 0.813 | 0.839 | 0.826 | 0.652 | 0.904 |
| *C.elegans* | 0.892 | 0.845 | 0.868 | 0.738 | 0.939 |
| *C.equisetifolia* | 0.697 | 0.715 | 0.706 | 0.412 | 0.778 |
| *D.melanogaster* | 0.892 | 0.870 | 0.881 | 0.762 | 0.943 |
| *F.vesca* | 0.921 | 0.913 | 0.917 | 0.834 | 0.968 |
| *H.sapiens* | 0.851 | 0.868 | 0.859 | 0.719 | 0.935 |
| *R.chinensis* | 0.902 | 0.732 | 0.817 | 0.649 | 0.914 |
| *S.cerevisiae* | 0.746 | 0.795 | 0.770 | 0.541 | 0.852 |
| *T.thermophile* | 0.946 | 0.774 | 0.860 | 0.731 | 0.920 |
| *Ts. SUP5-1* | 0.727 | 0.735 | 0.731 | 0.462 | 0.800 |
| *Xoc. BLS256* | 0.849 | 0.827 | 0.838 | 0.677 | 0.915 |
